# Vitamin D Status and Community-Acquired Pneumonia: Results from the Third National Health and Nutrition Examination Survey

**DOI:** 10.1371/journal.pone.0081120

**Published:** 2013-11-15

**Authors:** Sadeq A. Quraishi, Edward A. Bittner, Kenneth B. Christopher, Carlos A. Camargo

**Affiliations:** 1 Harvard Medical School, Boston, Massachusetts, United States of America; 2 Department of Anesthesia, Critical Care and Pain Medicine, Massachusetts General Hospital, Boston, Massachusetts, United States of America; 3 Department of Medicine, Brigham and Women's Hospital, Boston, Massachusetts, United States of America; 4 Department of Emergency Medicine, Massachusetts General Hospital, Boston, Massachusetts, United States of America; 5 Department of Epidemiology, Harvard School of Public Health, Boston, Massachusetts, United States of America; D'or Institute of Research and Education, Brazil

## Abstract

**Objective:**

To investigate the association between serum 25-hydroxyvitamin D [25(OH)D] level and history of community-acquired pneumonia (CAP).

**Patients and Methods:**

We identified 16,975 individuals (≥17 years) from the third National Health and Nutrition Examination Survey (NHANES III) with documented 25(OH)D levels. To investigate the association of 25(OH)D with history of CAP in these participants, we developed a multivariable logistic regression model, adjusting for demographic factors (age, sex, race, poverty-to-income ratio, and geographic location), clinical data (body mass index, smoking status, asthma, chronic obstructive pulmonary disease, congestive heart failure, diabetes mellitus, stroke, chronic kidney disease, neutropenia, and alcohol consumption), and season. Locally weighted scatterplot smoothing (LOWESS) was used to depict the relationship between increasing 25(OH)D levels and the cumulative frequency of CAP in the study cohort.

**Results:**

The median [interquartile range (IQR)] serum 25(OH)D level was 24 (IQR 18–32) ng/mL. 2.1% [95% confidence interval (CI): 1.9–2.3] of participants reported experiencing a CAP within one year of their participation in the national survey. After adjusting for demographic factors, clinical data, and season, 25(OH)D levels <30 ng/mL were associated with 56% higher odds of CAP [odds ratio 1.56; 95% confidence interval: 1.17–2.07] compared to levels ≥30 ng/mL. LOWESS analysis revealed a near linear relationship between vitamin D status and the cumulative frequency of CAP up to 25(OH)D levels around 30 ng/mL.

**Conclusion:**

Among 16,975 participants in NHANES III, 25(OH)D levels were inversely associated with history of CAP. Randomized controlled trials are warranted to determine the effect of optimizing vitamin D status on the risk of CAP.

## Introduction

Community-acquired pneumonia (CAP) is a common and potentially serious illness, which is associated with considerable morbidity and mortality [1]. Indeed, CAP is the leading infectious cause of death worldwide [2]. In the United States, it is estimated that there are over 4 million ambulatory care visits for CAP every year [3], resulting in excess of 1 million hospitalizations [4]. While the commercial availability of penicillin in the 1950s significantly improved survival in patients with pneumonia, advances in antimicrobial therapy since then have not resulted in further attenuation of mortality attributable to CAP [5]. Roughly 50,000 adults die from CAP each year in the United States [6]. Hospital and intensive care unit (ICU) admission rates continue to increase [7], and the annual direct and indirect cost associated with the care of patients with CAP exceeds $17 billion [5]. 

CAP is defined as an infection of the lung parenchyma that is not acquired in a healthcare setting [4]. Although it is thought to be primarily a result of pathogenic bacteria from the upper respiratory system or aspiration of material from the gastrointestinal tract, the exact pathophysiology is poorly understood [1,8]. Indeed, only a fraction of individuals in the general population with upper respiratory infections (URI) or those who aspirate progress to a fulminant pulmonary infection. In recent years, considerable interest has focused on circumstances that influence susceptibility to CAP, which has lead to the appreciation of independent risk factors such as smoking, asthma, and chronic obstructive pulmonary disease (COPD) [1,7]. Recently, in small or moderately sized cohorts of elderly, community-dwelling individuals, serum 25-hydroxyvitamin D [25(OH)D] levels were shown to be associated with the risk of CAP [9-11]. Given its central role in immune regulation, and its known association with various respiratory ailments (e.g. URI, asthma, COPD, tuberculosis) [12-14], vitamin D status may also be associated with CAP in the general adult population. Therefore, our goal was to investigate the association of 25(OH)D levels with history of CAP in a large, nationally-representative cohort of non-institutionalized individuals from the United States.

## Methods

The Third National Health and Nutrition Examination Survey (NHANES III) has been used extensively to report on the association of various biomarkers with major diseases. Conducted by the National Center for Health Statistics between October 18, 1988, and October 15, 1994, this cohort is regarded as a nationally-representative, cross-sectional sample of the non-institutionalized civilian population of the United States. We conducted a secondary analysis of this dataset. The Partners Human Research Committee granted an “exempt” status for the study.

### Data collection

Detailed survey methods, including sampling, interview, examination, laboratory measurements, ethics approval, and informed consent have previously been reported. In summary, the survey used a complex, stratified, multistage probability sample design. The NHANES III collected demographic information, biometric measurements, as well as data on health and nutrition on approximately 40,000 adults and children. The surveys were performed during scheduled in-home interviews, while physical examinations and laboratory testing were performed in either a mobile examination center or during a home visit. Blood samples collected during the examination were centrifuged, aliquoted, and stored at −70°C on-site. They were then shipped on dry ice to central laboratories, where they continued to be stored at −70°C until analysis. 25(OH)D levels were measured using a radioimmunoassay kit after extraction with acetonitrile (DiaSorin, Stillwater, MN) by the National Center for Environmental Health (Atlanta, GA).

### Data abstraction

We identified 20,039 individuals, 17 years and older, in the NHANES III dataset. From the household interview data file, we reported information on all participants related to self-reported: 1) age; 2) sex; 3) race; 4) poverty-to-income ratio (measure of socioeconomic status); and 5) geographical location. We also recorded current smoking status and self-reported current: 1) asthma; 2) chronic obstructive pulmonary disease (COPD); and 3) alcohol consumption. Additionally, we recorded self-reported history of congestive heart failure (CHF), diabetes mellitus (DM), and stroke. A diagnosis of COPD was based on responses to questions on emphysema and/or chronic bronchitis. From the physical examination data, we calculated body mass index (BMI). Furthermore, we used the laboratory data to document cases of neutropenia and to calculate estimated glomerular filtration rates (eGFRs) to assess for chronic kidney disease (CKD). To most accurately adjust for the effect of season on 25(OH)D levels, the date of the laboratory data collection was recorded. We limited our analysis to the 16,975 participants with reported 25(OH)D levels (primary exposure). The primary outcome (history of CAP) was based on the response to the following question: “During the past 12 months, have you had pneumonia?” 

### Statistical analysis

All statistical analyses were performed using Stata 12.0 (StataCorp LP, College Station, Texas). Using survey commands, we applied the recommended subsample weights for the interview plus examination data to account for unequal probabilities of selection and to accurately represent estimates for the population of the United States. All of the results are presented as weighted values. We calculated variance based on NHANES-provided masked variance units using the Taylor series linearization method. All reported P values are 2-tailed, with P<0.05 considered statistically significant. We calculated proportions with 95% confidence intervals (CIs) for demographic features and other factors thought to be related to CAP, overall and in the subset of participants with self-reported history of CAP within 12 months of the interview. 

For our primary analysis, we categorized serum 25(OH)D levels *a priori* as <30 ng/mL vs. ≥30 ng/mL. Based on existing guidelines [15], We sub-categorized 25(OH)D levels <30 ng/mL as <10 ng/mL, 10 to 19.9 ng/mL, and 20 to 29.9 ng/mL. To improve interpretability of the analysis, we converted age, BMI, geographic location, and season of blood draw into commonly used groupings. Specifically, we categorized geographic location into Northeast, Midwest, South, and West regions. Season of blood draw was categorized into Winter (December-February), Spring (March-May), Summer (June-August), and Fall (September-November). In addition, we dichotomized: 1) sex (female versus male); 2) race (non-white versus white); 3) poverty-to-income ratio (≤ federal poverty level versus federal poverty level); 4) smoking status; 5) CKD (eGFR <60 versus ≥60); 6) neutropenia (white blood cell count <3.5x10^3^ versus ≥3.5 x10^3^); and 7) alcohol consumption (≤30 versus >30 drinks per month). We also dichotomized self-reported histories of: 1) asthma; 2) COPD; 3) CHF; 4) DM; and 5) stroke. We determined unadjusted associations between risk factors and the outcome of CAP using the Pearson χ^2^ test for categorical variables and simple ordinal logistic regression for ordinal variables. To evaluate the independent association between serum 25(OH)D level and history of CAP, we created multivariable models by progressively adding covariates that might confound or alter the association of 25(OH)D with history of CAP. All adjusted odds ratios (ORs) for the variables in the models are reported with 95% confidence intervals (CIs). 

Locally weighted scatter plot smoothing (LOWESS) was used to graphically represent the association between 25(OH)D level and the cumulative frequency of CAP. LOWESS is a type of nonparametric regression, which summarizes the relationship between two variables in a fashion that initially relies on limited assumptions about the form or strength of the relationship [16]. The rationale and methods underlying the use of LOWESS for depicting the local relationship between measurements of interest across parts of their ranges are available elsewhere [17].

## Results

Characteristics of the weighted NHANES III sample are given in Table 1. The median age of the participants was 43 (IRQ 29-64) years; 53% were female and 68% were white. Overall, the median serum 25(OH)D level was 24 (IRQ 18-32) ng/mL. 2.1% (95%CI 1.9-2.3) of the overall cohort reported an episode of CAP within twelve months of their NHANES III interview. The proportions of participants with CAP, stratified by individual characteristics, are also given in Table 1. 

**Table 1:** Characteristics of the overall study cohort and in the subset with community acquired pneumonia.

	**Overall Study Cohort (Total Observations**)	**CAP - Number of Observations (% of overall study subsets)**	**P-value**
**25(OH)D**			
*<10 ng/mL*	641	20 (3.12)	<0.001
*10-19.9 ng/mL*	5110	85 (1.67)	
*20-29.9 ng/mL*	5931	144 (2.43)	
*≥30 ng/mL*	5293	72 (1.36)	
**Season**			
*Winter*	4555	84 (1.84)	0.15
*Spring*	5415	127 (2.35)	
*Summer*	4456	98 (2.20)	
*Fall*	5613	102 (1.82)	
**Age**			
*17-39 years*	8602	101 (1.17)	<0.001
*40-60 years*	4851	115 (2.37)	
*≥60 years*	6586	195 (1.77)	
**Sex**			
*Female*	10641	245 (2.30)	0.008
*Male*	9398	166 (1.77)	
**Race**			
*Non-white*	6310	87 (1.38)	<0.001
*White*	13729	324 (2.36)	
**Poverty ratio**			
*≤FPL*	4295	86 (2.00)	0.86
*>FPL*	15744	324 (2.06)	
**BMI**			
*<20 kg/m* ^*2*^	1470	49 (3.33)	0.004
*20-24.9 kg/m* ^*2*^	7163	134 (1.87)	
*25-29.9 kg/m* ^*2*^	6446	123 (1.91)	
*≥30 kg/m* ^*2*^	3567	82 (2.30)	
**Region**			
*Northeast*	29298	50 (1.71)	0.31
*Midwest*	3852	86 (2.33)	
*South*	8556	169 (1.98)	
*West*	4703	106 (2.25)	
**Smoking**			
*Yes*	4990	110 (2.20)	0.39
*No*	15049	301 (2.00)	
**Asthma**			
*>30 drinks per month*	1376	94 (6.83)	<0.001
*>30 drinks per month*	18663	317 (1.70)	
**COPD**			
*Yes*	1421	128 (9.00)	<0.001
*No*	18618	279 (1.52)	
**CHF**			
*Yes*	757	56 (7.40)	<0.001
*No*	19265	353 (1.83)	
**Diabetes mellitus**			
*Yes*	1614	70 (4.33)	<0.001
*No*	18410	341 (1.85)	
**Stroke**			
*Yes*	649	23 (3.54)	0.006
*No*	19393	388 (2.00)	
**CKD**			
*eGFR <60*	3388	92 (2.72)	<0.001
*eGFR ≥60*	12872	221 (1.72)	
**Neutropenia**			
*WBC <3.5x10^3^*	187	2 (1.07)	0.393
*WBC ≥3.5x10^3^*	16983	328 (1.93)	
**Alcohol consumption**			
*≤30 drinks per month*	1211	21 (1.73)	0.428
*>30 drinks per month*	18771	389 (2.07)	

CAP = Community-acquired pneumonia; 25(OH)D = 25-hydroxyvitamin D; FPL = federal poverty level; BMI – body mass index; COPD = chronic obstructive pulmonary disease; CHF = congestive heart failure; CKD = chronic kidney disease; eGFR = estimated glomerular filtration rate; WBC = white blood cell count. P-values are based on the chi-square test for categorical variables and on simple ordinal logistic regression for ordinal variables, with 2-tailed P<0.05 considered as statistically significant.

Compared with individuals with 25(OH)D levels ≥30 ng/mL, those with levels <30 ng/mL had a 56% higher adjusted odds of CAP (OR 1.56; 95%CI 1.17-2.07) within one year of the interview. Looking within the large group of participants with 25(OH)D levels <30 ng/ml, we found higher adjusted odds of CAP for those with levels <10 ng/mL (OR 2.25; 95%CI 1.26-4.01), 10-19.9 ng/mL (OR 1.26; 95%CI 0.89-1.79), and 20-29.9 ng/mL (OR 1.70; 95%CI 1.26-2.29). Other characteristics associated with CAP included age, race, asthma, COPD, CHF, and DM (Table 2).

**Table 2:** Multivariable model of factors associated with odds Adjusted odds ratios for covariates independently associated with the risk of community-acquired pneumonia in participants with 25-hydroxyvitamin D levels <30 ng/mL versus ≥30 ng/mL.

	**Odds Ratio (95% Confidence Interval)**
**25(OH**)**D (< 30 ng/ml vs. ≥30 ng/ml**)	**1.56 (1.17-2.07)**
Age (Years)	1.10 (1.01-1.19)
Race (White vs. Non-white)	1.64 (1.21-2.21)
Asthma	2.70 (2.00-3.66)
Chronic obstructive pulmonary disease	4.11 (3.09-5.47)
Congestive heart failure	1.87 (1.22-2.88)
Diabetes mellitus	1.53 (1.08-2.18)

25(OH)D = 25-hydroxyvitamin D.

**Figure 1:**
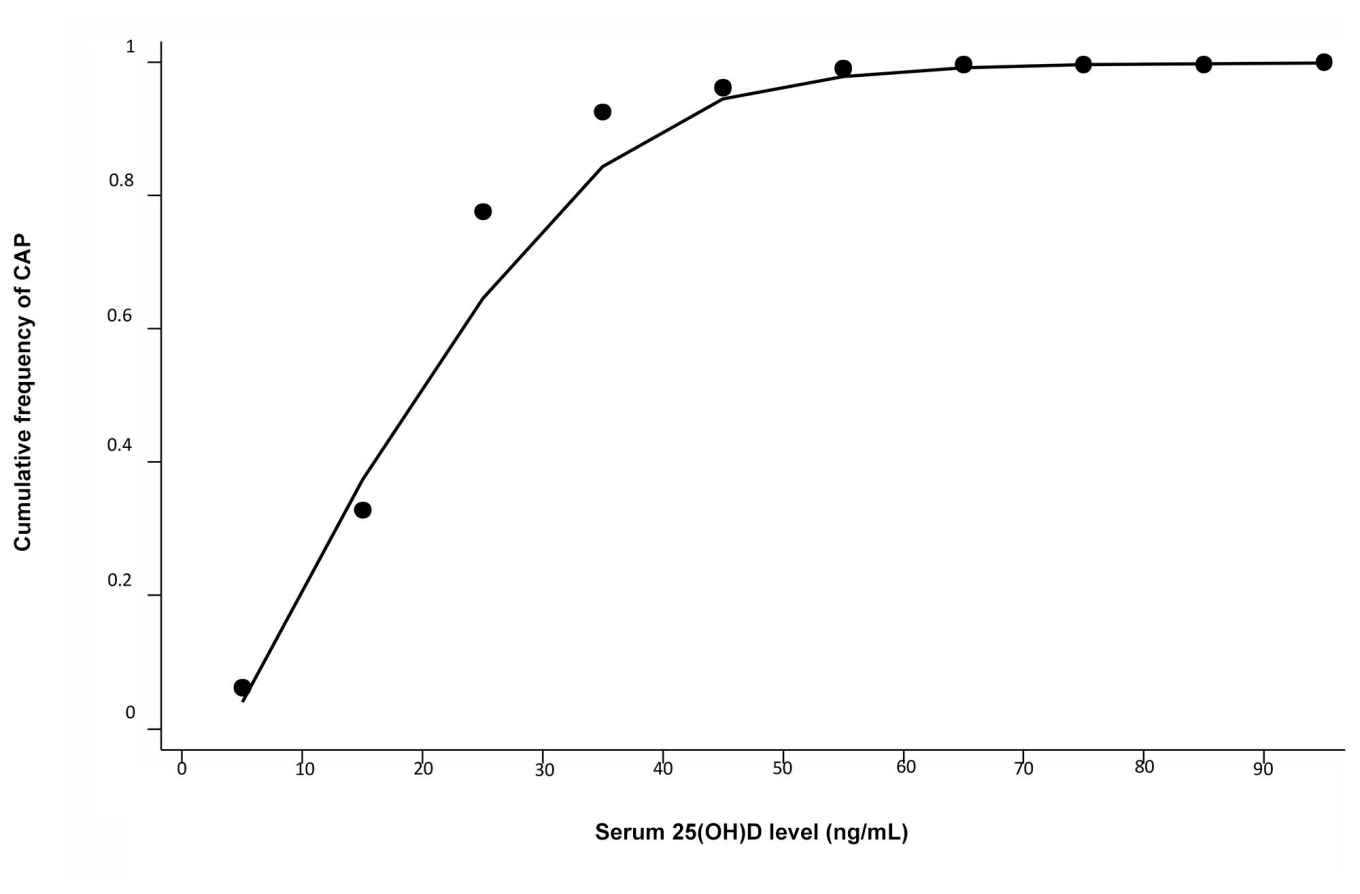
Locally weighted scatterplot smoothing (LOWESS) analysis to show the relationship between increasing 25-hydroxyvitamin D levels and the cumulative frequency of community-acquired pneumonia. CAP = community acquired pneumonia; 25(OH)D = 25-hydroxyvitamin D.

Although the subcategories of 25(OH)D suggested a rough dose-response association, LOWESS analysis showed a near linear relationship between 25(OH)D level and the cumulative frequency of CAP up to 25(OH)D levels around 30 ng/mL (Figure 1). Between 25(OH)D levels of 30 ng/mL and 60 ng/mL there was a progressive flattening of the curve. 

## Discussion

In this large nationally-representative study, we investigated whether 25(OH)D level was associated with history of CAP among adults in the United States. We demonstrated that 25(OH)D levels <30 ng/mL were indeed associated with a significant increase in the odds of CAP in the general population. While others have also hypothesized that vitamin D status may play an important protective role against various pulmonary diseases, our work provides important evidence to suggest that vitamin D supplementation may offer a novel approach to lowering the risk of CAP. Given the observational nature, and cross-sectional design of our current study, a causal inference about the effect of low vitamin D status and higher risk of CAP is not possible; however, the biological plausibility is undeniable. 

Recently, cells of the innate and adaptive immune system have been shown to express the vitamin D receptor [18]. Vitamin D appears to be necessary for interferon-γ dependent T cell responses to infection [19]. And, in low vitamin D states, dysfunctional macrophage activity becomes evident [20]. Vitamin D is also an important link between Toll Like Receptor (TLR) activation and antibacterial response [21]. Human macrophages stimulated by TLR induce: 1) vitamin D receptor expression [22]; 2) conversion of 25(OH)D to its most biologically active form of 1,25-dihydroxyvitamin D [23]; and 3) production of cathelicidin (LL-37), an endogenous antimicrobial peptide with potent activity against bacteria, viruses, fungi, and mycobacteria [24-26]. LL-37 is highly expressed at natural barrier sites (e.g. skin, gut, lungs) and may represent an important first-line of defense for the innate immune system [27]. 

In addition to the persuasive cellular, molecular, and biochemical data to support the relationship between 25(OH)D levels and immune function, associations of vitamin D status with the risk of various community-acquired and nosocomial infections are also evident. Indeed, 25(OH)D levels appear to influence the risk of URIs [28,29], bronchiolitis [30,31], and chronic sinusitis [32,33] in community dwelling adults and children. Moreover, emerging evidence suggests that vitamin D status may be associated with the risk of surgical site infections in post-operative patients [34] and with the risk of blood stream infections in hospitalized patients [35]. Taken altogether, these observations add to the growing body of evidence, which points to the critical role that vitamin D status may play in influencing host susceptibility to various infections. On the other hand, randomized, placebo-controlled, clinical trials (RCTs) have been less definitive [36,37]. While a handful of studies have attempted to investigate the effect of vitamin D supplementation on URIs [38-41], pneumonia [42,43], and mycobacterial pulmonary infections [44-46], RCTs demonstrating no effect have been criticized for the use of small sample sizes [38,40,44], low doses of vitamin D supplementation [38], intermittent dosing strategies that may have allowed for significant variation in 25(OH)D levels between doses [42,45], and for unexpectedly recruiting participants with 25(OH)D levels near 30 ng/mL at baseline [39].

Although the results of our present study are compelling, it is important to discuss potential limitations. Observational studies do not provide the highest level of clinical evidence, but they can highlight the existence or absence of a true effect and direct future research. Moreover, such cross-sectional studies may be limited by potential for confounding and the lack of a randomly distributed exposure. Despite adjustment for multiple potential confounders, there may still be residual confounding, which accounts for the observed differences in outcomes. Specifically, low vitamin D status may simply be a reflection of poor general health or suboptimal nutritional state, for which we are unable to fully adjust. We are also unable to fully adjust for lack of sun exposure (though we did control for the season during which blood draws were performed), physical activity, and immunization status. Although we are unable to directly control for cases of hospital-acquired pneumonia, the risk of including such individuals is small given that: 1) the NHANES III cohort represents an ambulatory, non-institutionalized group of participants; 2) we controlled for several major risk factors associated with low vitamin D status and high risk of requiring hospitalization; and 3) our regression analysis considered several covariates known to be associated with the severity of CAP. Given the confines of the NHANES III survey, a further limitation is that we were unable to control for the exact amount of time between the reported cases of CAP and the timing of blood draws. As such, we cannot rule out the possibility of reverse causation (i.e. low vitamin D status leads to CAP vs. CAP results in low vitamin D status). However, in non-hospitalized individuals, 25(OH)D levels tend to be relatively consistent over time (intra-person Pearson correlation coefficient of 0.70 at three years between blood draws following adjustments for age, race, and season) [47]. Nonetheless, vitamin D status may be influenced by critical illness [48], and therefore 25(OH)D levels may have been different at the time that participants developed CAP. And finally, the NHANES III dataset relies on a self reported history of CAP, which may be prone to inaccurate reporting. These and other potential issues will need to be addressed in future studies in order to replicate and extend our findings.

While the results of the current study indicate, that on aggregate, participants with 25(OH)D levels <30 ng/mL had a significantly higher risk of CAP compared to the group of participants with levels ≥30 ng/mL, we do note two peculiar observations. First, when individuals at the lower end of the 25(OH)D spectrum are further subdivided and compared to individuals with levels ≥30 ng/mL, as expected, those with levels <10 ng/mL had the highest odds of CAP (OR 2.25). However, the odds of CAP among participants with 25(OH)D levels of 10-19.9 ng/mL (OR 1.26) appeared to be lower than in those with levels of 20-29.9 ng/mL (OR 1.70) when both groups were compared to participants with 25(OH)D levels ≥30 ng/mL. While the incidence of CAP in participants with 25(OH)D levels of 10-19.9 ng/mL was significantly lower than that in the other two <30 ng/mL 25(OH)D subgroups (Table 1), a dose-response association remains apparent on the cumulative frequency LOWESS analysis (Figure 1). And second, while previous studies have suggested that ethnic minorities in the United States are at a greater risk for CAP than their white counterparts [49,50], our results differ, despite an intentional oversampling of non-whites in the NHANES III survey. Further studies are needed to address these unexpected observations.

## Conclusion

In summary, these data demonstrate that low 25(OH)D levels are strongly associated with history of CAP in a large nationally-representative cohort of non-institutionalized, adults in the United States. Longitudinal studies are required to confirm our findings and establish the mechanisms underlying these observations. If confirmed, high-quality randomized, controlled trials will be necessary to determine whether vitamin D supplementation therapy among adults with low vitamin D status may affect the incidence and severity of CAP in the general population.
