# Correction: Vitamin D Status and Community-Acquired Pneumonia: Results from the Third National Health and Nutrition Examination Survey

**DOI:** 10.1371/journal.pone.0091425

**Published:** 2014-03-07

**Authors:** The PLOS ONE Staff

There is an error in the first column of Table 1 under the sub-category of "Asthma."

**Table 1:** Characteristics of the overall study cohort and in the subset with community acquired pneumonia.

	Overall Study Cohort (Total Observations)	CAP - Number of Observations (% of overall study subsets)	P-value
			
**25(OH)D**			
*<10 ng/mL*	641	20 (3.12)	<0.001
*10-19.9 ng/mL*	5110	85 (1.67)	
*20-29.9 ng/mL*	5931	144 (2.43)	
*≥30 ng/mL*	5293	72 (1.36)	
			
**Season**			
*Winter*	4555	84 (1.84)	0.15
*Spring*	5415	127 (2.35)	
*Summer*	4456	98 (2.20)	
*Fall*	5613	102 (1.82)	
			
**Age**			
*17-39 years*	8602	101 (1.17)	<0.001
*40-60 years*	4851	115 (2.37)	
*≥60 years*	6586	195 (1.77)	
			
**Sex**			
*Female*	10641	245 (2.30)	0.008
*Male*	9398	166 (1.77)	
			
**Race**			
*Non-white*	6310	87 (1.38)	<0.001
*White*	13729	324 (2.36)	
			
**Poverty ratio**			
*≤FPL*	4295	86 (2.00)	0.86
*>FPL*	15744	324 (2.06)	
			
**BMI**			
*<20 kg/m^2^*	1470	49 (3.33)	0.004
*20-24.9 kg/m^2^*	7163	134 (1.87)	
*25-29.9 kg/m^2^*	6446	123 (1.91)	
*≥30 kg/m^2^*	3567	82 (2.30)	
			
**Region**			
*Northeast*	29298	50 (1.71)	0.31
*Midwest*	3852	86 (2.33)	
*South*	8556	169 (1.98)	
*West*	4703	106 (2.25)	
			
**Smoking**			
*Yes*	4990	110 (2.20)	0.39
*No*	15049	301 (2.00)	
			
**Asthma**			
*Yes*	1376	94 (6.83)	<0.001
*No*	18663	317 (1.70)	
			
**COPD**			
*Yes*	1421	128 (9.00)	<0.001
*No*	18618	279 (1.52)	
			
**CHF**			
*Yes*	757	56 (7.40)	<0.001
*No*	19265	353 (1.83)	
			
**Diabetes mellitus**			
*Yes*	1614	70 (4.33)	<0.001
*No*	18410	341 (1.85)	
			
**Stroke**			
*Yes*	649	23 (3.54)	0.006
*No*	19393	388 (2.00)	
			
**CKD**			
*eGFR <60*	3388	92 (2.72)	<0.001
*eGFR ≥60*	12872	221 (1.72)	
			
**Neutropenia**			
*WBC <3.5x10^3^*	187	2 (1.07)	0.393
*WBC ≥3.5x10^3^*	16983	328 (1.93)	
			
**Alcohol consumption**			
*≤30 drinks per month*	1211	21 (1.73)	0.428
*>30 drinks per month*	18771	389 (2.07)	
			

CAP  =  Community-acquired pneumonia; 25(OH)D  =  25-hydroxyvitamin D; FPL  =  federal poverty level; BMI – body mass index; COPD  =  chronic obstructive pulmonary disease; CHF  =  congestive heart failure; CKD  =  chronic kidney disease; eGFR  =  estimated glomerular filtration rate; WBC  =  white blood cell count. P-values are based on the chi-square test for categorical variables and on simple ordinal logistic regression for ordinal variables, with 2-tailed P<0.05 considered as statistically significant.

Additionally, there is an error in the title for Table 2.

**Table 2:** **Multivariable model of factors independently associated with the risk of community-acquired pneumonia (with adjusted odds ratios) in participants with 25-hydroxyvitamin D levels <30 ng/mL versus ≥30 ng/mL.**

	Odds Ratio (95% Confidence Interval)
	
**25(OH)D (< 30 ng/ml vs. ≥30 ng/ml)**	**1.56 (1.17-2.07)**
Age (Years)	1.10 (1.01-1.19)
Race (White vs. Non-white)	1.64 (1.21-2.21)
Asthma	2.70 (2.00-3.66)
Chronic obstructive pulmonary disease	4.11 (3.09-5.47)
Congestive heart failure	1.87 (1.22-2.88)
Diabetes mellitus	1.53 (1.08-2.18)
	

25(OH)D  =  25-hydroxyvitamin D.
